# Temperature Rise during Primer, Adhesive, and Composite Resin Photopolymerization of a Low-Shrinkage Composite Resin under Caries-Like Dentin Lesions

**DOI:** 10.5402/2012/198351

**Published:** 2012-12-24

**Authors:** Sayed-Mostafa Mousavinasab, Maryam Khoroushi, Mohammadreza Moharreri

**Affiliations:** ^1^Torabinejad Dental Research Center and Department of Operative Dentistry, School of Dentistry, Isfahan University of Medical Sciences, Isfahan 81746-73461, Iran; ^2^Dental Materials Research Center and Department of Operative Dentistry, School of Dentistry, Isfahan University of Medical Sciences, Isfahan 81746-73461, Iran; ^3^Department of Operative Dentistry, School of Dentistry, Isfahan University of Medical Sciences, Isfahan 81746-73461, Iran

## Abstract

*Objective*. This study evaluated temperature rise of low-shrinkage (LS) self-etch primer (P), LS self-etch adhesive (A), and P90 silorane-based composite resin systems, photopolymerized under normal and artificially demineralized dentin. *Methods*. Forty 1.5 mm-thick dentin discs were prepared from sound human molars, half of which were demineralized. Temperature rise was measured during photopolymerization using a K-type thermocouple under the discs: 10 s and 40 s irradiation of the discs (controls/groups 1 and 2); 10 s irradiation of primer (P), 10 s irradiation of adhesive (A), 40 s irradiation of P90 without P and A, and 40 s irradiation of P90 with P and A (groups 3 to 6, resp.). The samples were photopolymerized using an LED unit under 550 mW/cm^2^ light intensity. Data was analyzed using repeated measures ANOVA and paired-sample *t*-test (*α* = 0.05). *Results*. There were no significant differences in temperature rise means between the two dentin samples for each irradiation duration (*P* > 0.0001), with significant differences between the two irradiation durations (*P* > 0.0001). Temperature rise measured with 40 s irradiation was significantly higher than that of 10 s duration for undemineralized and demineralized dentin *P* < 0.0001). *Conclusions*. Light polymerization of P90 low-shrinkage composite resin resulted in temperature rise approaching threshold value under artificially demineralized and undemineralized dentin.

## 1. Introduction

Exothermic photopolymerization reaction of resin-based restorative materials and the heat produced by light-curing units (LCUs) might irritate the pulp due to an increase in temperature in the tooth cavity [1, 2]. Various factors, including the intensity of the light [3], the chemical composition of the restorative material [4, 5], heat conduction properties of composite resins [6], the depth of the cavity or the thickness of the restoration [6, 7], and irradiation duration [8, 9], might influence the extent of temperature rise during photopolymerization. 

Studies have shown that the heat generated during restorative procedures might have a detrimental effect on dental pulp. There is still controversy over the 5.5°C threshold temperature rise for irreversible changes in the dental pulp [10, 11].

Dentin has been reported to have a low thermal conductivity; however, the risk of irreversible pulp damage is greater in deep cavities with minimal residual dentin thickness, in which there is a concomitant increase in tubular surface area [12]. A large number of *in vitro* studies have been carried out to determine temperature rise during light-curing procedures of resin-based restorative materials; these studies have predominantly used ground or non-carious dentin substrates [13–16]. However, in most clinical situations non-carious dentin is not encountered [17], and carious dentin is generally restored. Carious dentin characteristically consists of infected and affected layers; the affected dentin layer is not usually removed during restorative procedures [18, 19]. Therefore, a layer of caries-affected dentin remains on the cavity floor after removal of a carious lesion and preparation of the cavity for an adhesive restorative procedure [19, 20].

Only a limited number of studies have been carried out in an attempt to evaluate temperature rise during the photopolymerization procedures of resin-based restorative materials beneath sound and carious dentin [21, 22]. Fanbunda and colleague [21] reported a significantly higher thermal conductivity for carious dentin compared to sound dentin, that is, carious dentin has less capacity to form a thermal barrier against heat. Tosun et al. [22] compared the temperature rise under sound and caries-affected primary tooth dentin during photopolymerization of two adhesives, including AdheSE Bond and One Step Plus, and two resin-based restorative materials, including Compoglass F and Aelite LS. They reported a temperature rise beyond 5.5°C threshold during photopolymerization of adhesive materials under caries-affected primary tooth dentin. Moreover, adhesive systems induced a higher temperature rise during photopolymerization in comparison to resin-based restorative materials. The structural differences between sound and caries-affected primary tooth dentin had an influence on temperature rise during photopolymerization of resin-based materials.

Filtek LS (3M ESPE, St. Paul, MN, USA), a new silorane-based RBC resin, has been developed in an attempt to minimize polymerization shrinkage. Its siloxane oxirane resin molecule is light-cured through a cationic ring-opening reaction after an interaction between camphorquinone, iodonium salts, and electron donors. The manufacturer has recommended that the light-curing procedure should exceed 20 seconds because this is the minimum duration of time for the activation of the initiator [23]. Furthermore, based on manufacturer's instructions, there are separate 10 s photopolymerization steps for the LS primer and LS adhesive in this system (Table 1) [23].

Optical pyrometry studies have revealed that cationic ring-opening polymerization of oxiranes is highly exothermic, with temperature increases from the ambient temperature to >100°C in a few seconds [24]. It has been postulated that the amount of heat generated during cationic photopolymerization has a direct relationship with the number of photogenerated initiating species in the system [25].

The aim of this *in vitro* study was to evaluate temperature fluctuations under sound and artificially demineralized dentin during light polymerization of the LS self-etch primer (P) and adhesive (A) and the silorane-based composite resin (P90). The null hypothesis was that the temperature rise induced by each photopolymerization step of LS primer, LS bond and LS composite under undemineralized dentin (control) would not be different from that under artificially demineralized dentin. 

## 2. Materials and Methods

Table 1 presents the chemical compositions and instructions for use of Filtek P90 primer, adhesive, and restorative material used in the present study. Shade A3 of the material was photopolymerized using Bluephase light source (Bluephase, Ivoclar Vivadent, Schaan, Liechtenstein) at 550 mW/cm^2^. The light output of the polymerizing unit was checked before each procedure using a radiometer (Curing Radiometer, Kerr, Orange, CA, USA).

### 2.1. Specimen Preparation

The research protocol was approved by the Human Ethics Committee of the School of Dentistry, Isfahan University of Medical Sciences. Forty extracted sound human third molars were used in the present study within three months of extraction.

**Figure 1:**
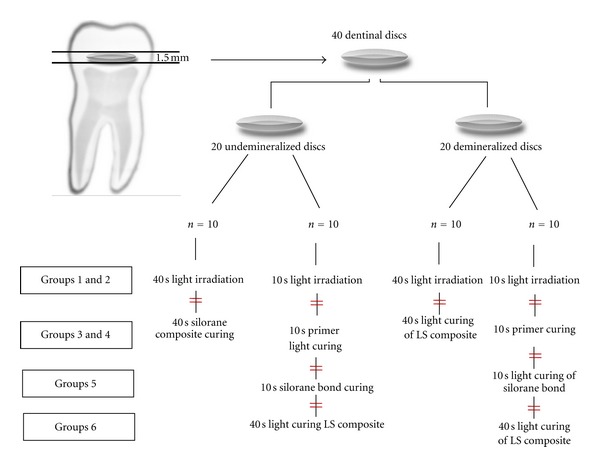
Schematic view of the different steps for preparation of the dentinal discs and different groups. Signs “=” show the steps which the temperature rise under the each disc has been recorded using thermocouple.

Dentin discs measuring 1.5 mm in thickness were prepared from the deep dentin layers of 40 extracted third molars. First, each tooth was mounted in epoxy resin (Triplex Acryl, Ivoclar Vivadent, Schaan, Liechtenstein); then sections were prepared parallel to the long axis of each tooth with a low-speed diamond saw (Servocut, M-300, Switzerland) under copious water irrigation. Perpendicular cuts were made to prepare dentin disks measuring 1.5 mm (Figure 1). The surfaces of the samples were wet-ground using 320- and 400-grit SIC abrasive papers under water cooling to prepare flat dentin surfaces.

Half of the samples were placed in a demineralizing solution to prepare artificially demineralized dentin samples resembling caries-like dentin lesions; each dentin sample was placed in 20 mL of a demineralizing solution for 48 hours at 23°C [26]. The chemical composition of the solution was as follows: CaCl_2_, 0.002 M/L; KH_3_PO_4_, 0.002 M/L; glacial acetic acid, 0.002 M/L, with a pH value of 4.3 [26]. Finally, the samples were stored in water to prevent dehydration until used for the purpose of the study.

### 2.2. Temperature Measurements

The ambient temperature was 23 ± 1°C; the temperature of a 40 s irradiation procedure of the unit, without any dentin or restorative material samples, was recorded at 18.19°C. Temperature rise in the dentin samples was measured prior to photopolymerization procedures using 10 s and 40 s test runs so that it would be possible to assess whether structural variables of dentin samples would affect temperature variations. 

The mean of temperature rise was measured during 10 s (group 1) and 40 s (group 2) irradiation durations with the LED light-curing unit under the undemineralized and demineralized dentin samples. 

In order to measure temperature rise during photopolymerization of the LS system primer (LS Primer, 3M ESPE) (groups 3), the material was applied on undemineralized and demineralized dentin samples and cured according to manufacturers' instructions (Table 1). The same steps were followed for the LS system adhesive (groups 4) (Figure 1), that is, the LS system adhesive was applied on the dentin samples and light-cured LS primer and photopolymerized for 10 seconds. Temperature variations were recorded for all the demineralized and undemineralized dentin samples (*n* = 10) (Figure 1).

After photopolymerization of the primer and adhesive resin, the temperature rise was measured. Subsequently, the central cylinder was filled with 2 mm of the Filtek P90 composite resin and light-cured for 40 seconds (Table 1) (groups 5). Furthermore, for 10 undemineralized and 10 demineralized dentin samples, a 2 mm thick sample of Filtek LS, without primer and adhesive, was light-cured for 40 s; then temperature rise was measured beneath demineralized and undemineralized dentin samples (groups 6).

**Figure 2:**
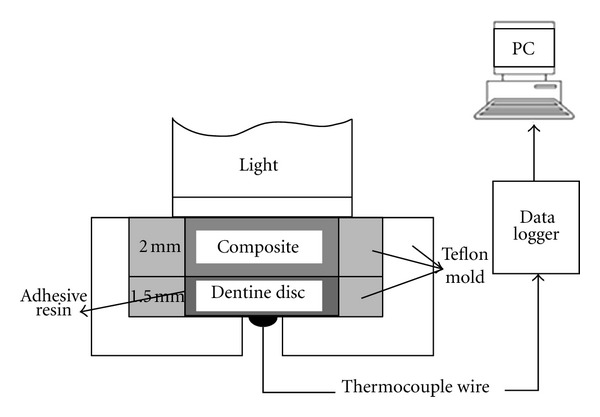
Schematic view of the temperature rise measurement during polymerization of the materials under the dentin discs.

For all the study groups, temperature rise was measured beneath demineralized and undemineralized dentin samples. To this end, a Teflon mold was used to support the dentin sample and the resin restorative material (Figure 2). Tip of the light conductor of the curing unit was placed on the LS primer and adhesive with a 2-mm distance in between; no space was left between the LS resin materials and the tip with the use of a celluloid matrix. A K-type thermocouple wire with a diameter of 0.1 cm (Standard, ST-8891E, Taiwan) was connected to a data logger (Standard, ST-8891E, Taiwan) during the light-curing procedure of the LS primer, LS adhesive and Filtek LS composite resin restorative material (Figure 2).

Sampling rate of data logger was adjusted at one sample per second, starting with light-curing for 120 seconds until the temperature began to decrease. Data, presented in graphic forms, was monitored real-time and recorded in a computer.

Light output of the curing unit was evaluated prior to each procedure using an LED radiometer (LED Radiometer, Kerr, Orange, CA, USA). The intensity of light during the test procedures was adjusted on a low intensity of 500 mW/cm^2^. Subsequent to light output evaluation, the test was repeated for 10 dentin samples in each group (Figure 1). The difference between the baseline temperature and the highest temperature recorded was calculated and registered. Temperature means and standard deviations were obtained for each material with demineralized and undemineralized dentin samples. There were ten samples in each group. Data was analyzed using SPSS software Ver. 13.5. Statistical significance was defined at *α* = 0.05. Data were analyzed by repeated measures ANOVA. Paired sample *t*-test was used to evaluate differences between the study groups.

## 3. Results

**Figure 3:**
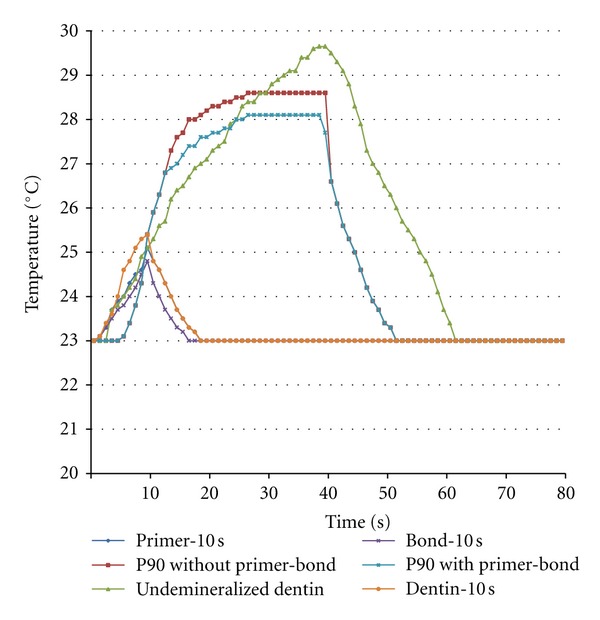
Temperature changes during primer, adhesive, and composite resin photopolymerization of silorane-based low-shrinkage composite under undemineralized dentin.

**Figure 4:**
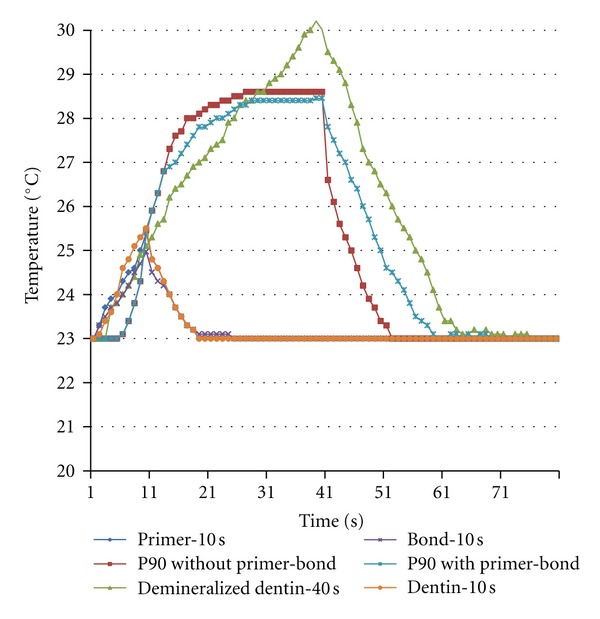
Temperature changes during primer, adhesive, and composite photopolymerization of silorane-based low-shrinkage composite under demineralized dentin.

Table 2 presents temperature variation means during the photopolymerization procedures of the 6 study groups. Table 2, and Figures 3 and 4 present temperature fluctuations during each photopolymerization step of LS primer, LS adhesive and LS composite resin beneath the undemineralized and demineralized dentin samples, respectively.

The mean temperature rise values during photopolymerization for 10 s beneath undemineralized and demineralized dentin samples without any material (groups 1) were 2.39 ± 0.60°C and 2.47 ± 0.40°C, respectively. The mean temperature rise values during photopolymerization for 40 s were 6.26 ± 1.10°C and 7.02 ± 0.43°C with undemineralized and demineralized dentin samples, respectively. There were no significant differences in temperature rise means between the two dentin disc samples for each irradiation duration (*P* > 0.05). However, significant differences were revealed between the two irradiation durations (*P* < 0.0001) (Table 2). On the other hand, ANOVA and paired *t*-test, which were used to determine differences in temperature rise between the groups within the materials and durations, showed that all temperature rise values measured with 40 s duration were significantly higher than those of 10 s duration for both undemineralized and demineralized dentin samples (*P* < 0.0001) (Table 2).

Although the mean of temperature rise during 40 s light-curing procedure of Filtek LS without primer and adhesive (groups 6) was higher than that in group 5, in which 2-mm-thick Filtek LS was applied subsequent to the application and curing of primer and adhesive according to the manufacturer's instructions, there were no significant differences between the groups above under both undemineralized and demineralized dentin (*P* > 0.05). The maximum temperature rise was recorded during photopolymerization of Filtek P90 under demineralized dentin samples without application and curing of primer and adhesive (6.25 ± 1.02°C), followed by that recorded in the corresponding group with undemineralized dentin sample (5.58 ± 0.86°C). 

## 4. Discussion

Photopolymerization of dental adhesives and resin-based composite resins increases dentin temperature through exothermic resin polymerization process and the energy absorbed during light-curing. The rate of exothermic polymerization reaction and maximum temperature rise during the process is proportional to the irradiance of the light-curing unit, chemical structure of the adhesive, and/or the composite resin and the light conduction properties of composite resin [27–29].

Although dentin has a low thermal conductivity, the risk of pulp damage is high in deep composite restorations because dentinal tubules are more numerous and denser in deep cavities. As a result, irritation of the pulp due to heat depends on the extent and duration of temperature rise [30–32].

The results of the present study in relation to the comparison of groups 1 and 2, in which only sound and demineralized dentin samples without any changes were irradiated, showed that a 10 s irradiation (group 1) resulted in a temperature rise of 2.39°C and 2.47°C in undemineralized and demineralized dentin samples, respectively; a 40 s irradiation (group 2) increased dentin sample temperatures up to 6.26°C and 7.02°C, respectively, demonstrating statistically significant differences between the two groups. 

Based on the results of several studies [29–33], the irradiation intensity and the duration of curing are the most important factors influencing temperature fluctuations, consistent with the results of the present study.

 There is consensus that temperature rise due to certain dental procedures threatens pulp vitality [34]. It has also been postulated that visible light polymerization units might increase temperatures within the pulp chamber, irritating the pulp [35]. In the present study, in groups 3 to 5, Filtek P90 composite resin was applied by exactly following the manufacturer's instructions, that is, in each of the steps involving the application of primer, adhesive, and the LS composite resin the temperature rise was accurately measured through normal and demineralized dentin.

An animal study by Zach and Cohen resulted in determination of a threshold temperature for irreversible pulp injury during application of external heat to a sound tooth: a 5.5°C intrapulpal temperature rise gave rise to necrosis in 15% of the pulps [10].

In this *in vitro* study temperature variations were evaluated under demineralized and undemineralized dentin samples during the photopolymerization of primer, adhesive and composite resin of a low-shrinkage silorane system using an LED light-curing unit. The numeric values of temperature rise after photopolymerization of each material–primer, adhesive and silorane-based composite resin–were higher with demineralized dentin compared to normal dentin samples; however, the mean values were near the threshold only during photopolymerization of the LS composite resin for 40 s.

Previous studies have shown that the extent of temperature depends on two factors: the exothermic polymerization reaction of composite resin during photopolymerization and the energy output of the light-curing unit [36–38]. The results of the present study supported the hypothesis that longer photopolymerization times result in significantly greater temperature rise compared to shorter irradiation times.

It has already been reported in relation to conventional methacrylate-based composite resins that the temperature rise decreases as the resin filler content of a dental material increase because less resin is available for polymerization. Fillers are chemically inert and do not contribute to an increase in the heat of reaction. However, filler temperature increases along with that of the matrix. Therefore, a considerable proportion of the energy which might otherwise increase the temperature of the resin matrix is absorbed and dissipated by the filler. In this context, fillers are active phases with a moderate role [32, 38].

A silorane-based resin composite has recently been introduced. The term “silorane” is derived from its chemical composition of siloxanes and oxiranes [39]. The combined properties of siloxanes and oxiranes result in a composite resin which is claimed to be biocompatible, hydrophobic and low-shrinkage [39]. The new resin matrix has a specific two-part self-etching primer adhesive. The primer component of the adhesive consists of hydrophilic methacrylate-based resins similar to those of other adhesive systems [39]; however, the hydrophobic adhesive bond, which has specially been designed to achieve compatibility with the newly developed silorane restorative resin [39], has been claimed to have lower polymerization stress and shrinkage. 

Based on manufacturer's recommendations, the primer, the adhesive and the composite resin should be cured for 10, 10 and 40 s, respectively [23]. Groups 3 to 5 were designed for each of the procedural steps of material application for more accurate evaluation of temperature variations through normal and demineralized dentin samples. Based on the results, LS primer/adhesive and LS restorative material exhibited different maximum temperature rise values, with statistically significant differences, which might be attributed to differences in polymerization times (10 s versus 40 s) and also to differences in resin contents.

A number of previous studies have shown that adhesive resins, which are unfilled materials, exhibit greater temperature variations compared to resin-containing restorative materials [28, 38]. Comparison of groups 3 and 4 with group 1 in the present study showed a slight protective effect of the adhesive layer. It should also be pointed out that adhesive resins absorb heat energy during photopolymerization as they confer thermal insulation to the underlying dentin and pulpal tissue during the process [28].

In the present study, the mean temperature rises were recorded during photopolymerization of LS primer (2.34°C), LS Bond (1.98°C) and Filtek LS composite (5.45°C) beneath demineralized dentin samples. The values for the primer and adhesive were much lower than the temperature threshold for irreversible pulp injury; however, it was near the threshold for Filtek LS composite resin. Fortunately, temperatures are modified by the circulation in the pulp chamber and fluid movements in dentinal tubules in clinical situations [22, 30]. Moreover, periodontal tissues can dissipate heat *in vivo*, further decreasing intrapulpal temperature [22]. Therefore, the hypothesis of the study was confirmed for the LS self-etch primer (P) and adhesive (A) systems and was rejected for the silorane-based composite resin system (P90) because the highest values of temperature rise during photopolymerization of the LS system was recorded in groups 5 and 6, especially with the demineralized dentin, in which irradiation duration was 40 s, which was near the critical threshold value.

This observation is consistent with the results of studies by Shortall and Harrington [40] and da Silva et al. [38] concluding that the light attenuating ability of the material exceeds the effect of exothermic polymerization reaction at the base of the cavity floor. Although dentin has a relatively low thermal conductivity, there is a higher risk for pulp injury in deep cavities in which there is a thin layer of residual dentin, with increased permeability of dentin tubules. Knezevic et al. [36] reported that the temperature rise increases as the material's thickness decreases.

Based on the results of a study by Lloyd and Brown [34], the rate of exothermic photopolymerization reaction is directly proportional to the inorganic content of composite resin. Therefore, as the inorganic content decreases, the organic content increases and as a result, the exothermic reaction is stronger. The results of the present study showed that the Filtek LS composite resin without LS primer and/or LS adhesive (G5) was not statistically different in temperature change from the same composite resin with LS primer and LS adhesive due to similar content, thickness, and curing protocol.

In the present study, temperature rise was higher in the primer compared to the adhesive after photopolymerization. It appears the light-cured primer plays the role of an insulating layer for the heat produced as a result of photopolymerization of the adhesive layer to some extent. In this context, comparison of groups 5 and 6 showed the effect of light-cured primer and adhesive layers on the decrease of heat transfer through both types of dentin with LS composite resin. It appears the light-cured primer and adhesive layers function as an insulating layer for the heat generated as a result of photopolymerization of composite resin. Evaluation of temperature changes during polymerization of each LS system layer (Figures 3 and 4) confirm the mean results achieved.

Previously, Miletic et al. showed that if the light output energy during photopolymerization remains constant for all resin-based composite resins, including silorane-based composite resin, a substantially different heat generation pattern would be observed with Filtek LS, which might be attributed to a different photopolymerization reaction. Filtek LS is a siloxane- and oxirane-based composite resin, which polymerizes through a cationic ring-opening reaction [28]. This reaction takes place in the oxirane component and is initiated by a photochemical reaction during which camphorquinone excited by light energy, interacts with iodonium salts and electron donors to yield cations as propagating active centers. In brief, in the present study comparison of groups 5 and 6 showed the effect of light-cured primer and adhesive layers on a decrease in light conductivity through both types of dentin [28].

In the present study an LED light-curing unit with low intensity mode of 550 mW/cm^2^ was used. Some studies [28, 38] have shown that QTH units with higher light intensities result in more temperature variations with dimethacrylate-based composite resins [32]; in this context, future studies should investigate low-shrinkage composite resins. Although the present study showed mean temperature rises of 5.58°C and 6.25°C under the undemineralized and demineralized dentin, respectively, it can be suggested that this range of temperature rise might induce pulp injury because it has been reported that a temperature rise in the order of 5.5°C during photopolymerization of dental restorative materials is the threshold to avoid irreversible changes in the pulp [10]. However, some other studies have shown that even higher temperature rises cannot induce irreversible pulp damage [41]. Therefore, there is controversy over the critical temperature threshold beyond which irreversible pulp damage occurs; however, it can be recommended that the pulp temperature should be kept as low as possible during composite resin photopolymerization to avoid any irreversible pulp injury [38].

Comparison of temperature changes in different groups of the present study, particularly in the presence of demineralized dentin, which is very similar to clinical situations, shows that dental practitioners should be aware of the risk of thermal insults to the pulp as a result of photopolymerization of resin-containing restorative materials, especially when caries-affected deep dentin is involved for longer periods. In such conditions, a simple but very effective method for pulp protection is to use an effective cement base or lining material to reduce the intrapulpal temperature rise during light polymerization of composite resin [22].

## 5. Conclusions

Within the limitations of this *in vitro* study, the following conclusions were drawn.

40 s light-curing of Filtek LS exhibited a significantly different heat generation mode and significantly greater temperature variations beneath 1.5 mm thick samples of demineralized and undemineralized dentin samples compared to 10 s light-curing of each of the LS primer and adhesive. Light-cured self-etching LS system primer/adhesive functions as a heat insulation layer during photopolymerization of the Filtek LS restorative material.

The structural differences between undemineralized and demineralized dentin samples did not significantly influence temperature rise during photopolymerization of the low-shrinkage composite resin under study.

There were no significant differences between the temperature rises as a result of photopolymerization of the LS system self-etch adhesive primer/adhesive under demineralized and undemineralized dentin samples.

Temperature rise during photopolymerization of Filtek LS restorative material exceeded 5.5°C under demineralized dentin samples, which should be evaluated in future studies.

## Figures and Tables

**Table 1:** Materials used in the study, their compositions and sources.

Material	Composition	Manufacturer
LS system adhesive self-etch primer (pH: 2.7)	Phosphorylated methacrylates, Vitrebond copolymer, bis-GMA, HEMA, water, ethanol, silane-treated silica filler, initiators, stabilizers	3M ESPE, St. Paul, MN, USA
LS system adhesive self-etch bond	Hydrophobic dimethacrylate, phosphorylated methacrylates, TEGDMA, silane-treated silica filler, initiators, stabilizers	3M ESPE, St. Paul, MN, USA
Filtek LS	Silorane resin, initiating system, quartz filler, yttrium fluoride, stabilizers, pigments	3M ESPE, St. Paul, MN, USA

bis-GMA: Bisphenol A glycol dimethacrylate; HEMA: 2-hydroxyethyl methacrylate; TEGDMA: triethyleneglycol dimethacrylate.

**Table 2:** Temperature rise values (mean ± SD) during photopolymerization under demineralized and undemineralized dentin disks (*n* = 10).

Groups	Groups definitions	Mean ± SD (°C)	
		Undemineralized dentin	Demineralized dentin
1	Δ*T* Dentin (10 s light curing)	2.39 ± 0.60^aA^	2.47 ± 0.40^aA^
2	Δ*T* Dentin (40 s light curing)	6.26 ± 1.10^aB^	7.02 ± 0.43^aB^
3	Δ*T* LS System adhesive primer (10 s light curing)	2.14 ± 0.31^aA^	2.34 ± 0.27^aA^
4	Δ*T* LS System adhesive bond (10 s light curing)	1.83 ± 0.17^aA^	1.98 ± 0.24^aA^
5	Δ*T* Filtek LS with primer and adhesive (40 s light curing)	5.14 ± 0.62^aB^	5.45 ± 0.49^aB^
6	Δ*T* Filtek LS without primer and adhesive (40 s light curing)	5.58 ± 0.86^aB^	6.25 ± 1.02^aB^

Means followed by different letters show statistical differences: (*α* = 0.05).

Lower case letters: comparison of dentin discs at each procedure (row).

Capital letters: comparison of each photopolymerization step for each kind of dentin (column).

Δ*T*: temperature change.
